# Supplementary material for: Binding properties of sulfur to enable solvent-free fabrication of high-performance polymer-free sulfur-carbon positive electrodes
Source: Nat Commun. 2026 Feb 4;17:2360. doi: 10.1038/s41467-026-69097-6 (PMC12979846; doi:10.1038/s41467-026-69097-6)
Supplement: Supplementary file 1 — Supplementary Information [file 41467_2026_69097_MOESM1_ESM.pdf]

Supplementary Information

Binding properties of sulfur to enable solvent-free fabrication of high-performance polymer-free sulfur-carbon positive electrodes

Yuhui An,[1,$] Kyungbae Kim,[1,$] Yun-Jeong Lee,[2] Soyeon Ko,[3] Faizan Ejaz,[4] Yongming Liu,[4] Beomjin Kwon,[4] Seung-Ho Yu,[2,5,*] and Yoon Hwa[6,*]

[1]Materials Science and Engineering, Fulton School of Engineering, Arizona State University, Tempe, AZ, 85287, USA

[2]Department of Chemical and Biological Engineering, Korea University, 145 Anam-ro, Seongbuk-gu, Seoul, 02841, Republic of Korea

[3]Chemical Engineering, Fulton School of Engineering, Arizona State University, Tempe, AZ, 85287, USA

[4]Mechanical and Aerospace Engineering, Fulton School of Engineering, Arizona State University, Tempe, AZ, 85287, USA

[5]Department of Battery-Smart Factory, Korea University, 145 Anam-ro, Seongbuk-gu, Seoul, 02841, Republic of Korea

[6]School of Electrical, Computer and Energy Engineering, Arizona State University, Tempe, AZ, 85287, USA

$An, Y. and Kim, K. contributed equally to this work.

*Corresponding Authors:
E-mail: seunghoyu@korea.ac.kr (Yu, S.-H.) and Yoon.Hwa@asu.edu (Hwa, Y.)

Supplemental Figures

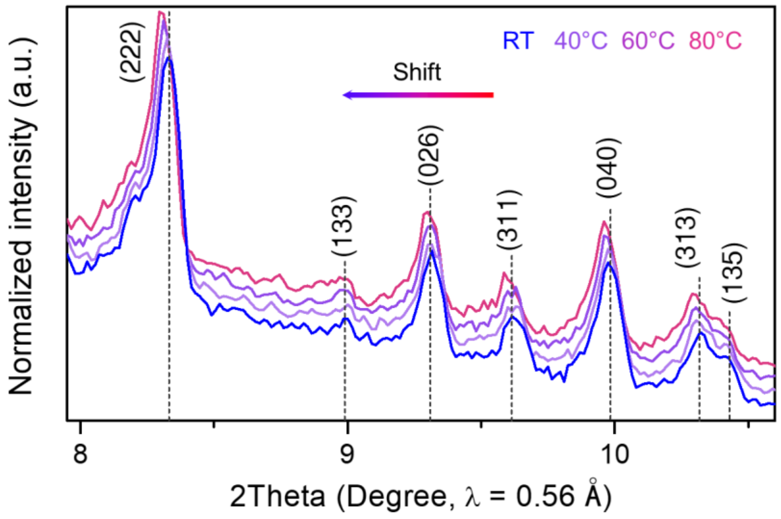

Figure S1. Operando XRD patterns of elemental sulfur during heating. Enlarged view of Fig. 1a, focusing on the 2θ range between 8.0° and 10.6°. The temperature was elevated from room temperature (RT) to 80 °C at a ramping rate of 1 °C min^{-1}.

As shown in the XRD pattern (Fig. 1a and S1), the (222) plane of the sulfur crystal shifts towards a lower 2θ angle, particularly above 60 °C (diffraction peak positions listed in Table S1), indicating thermal lattice expansion.

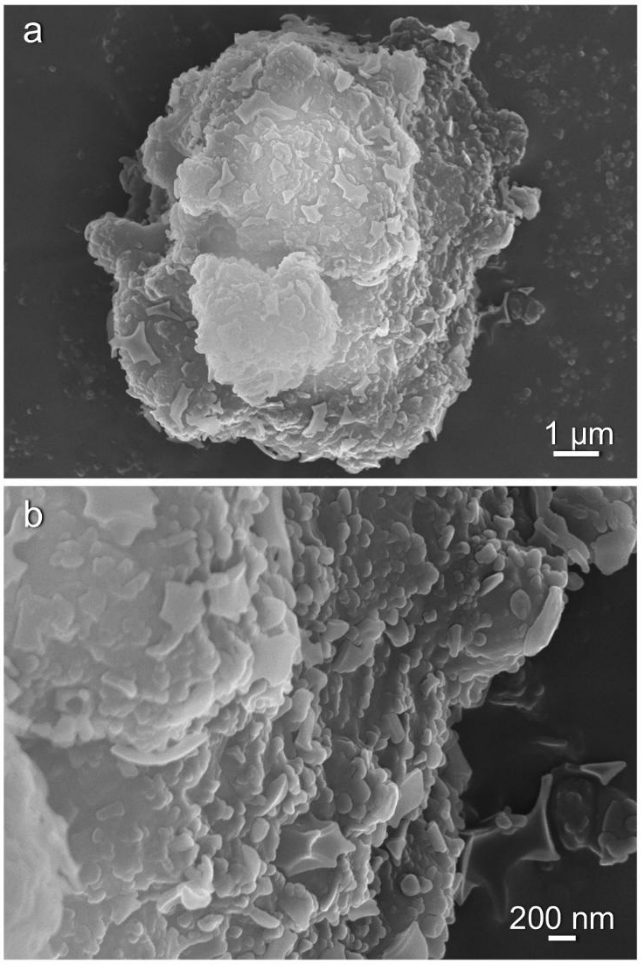

Figure S2. Scanning electron microscope images of commercial elemental sulfur powder used in this study. a) low-magnification view of a representative sulfur particle. b) high-magnification image of the same particle.

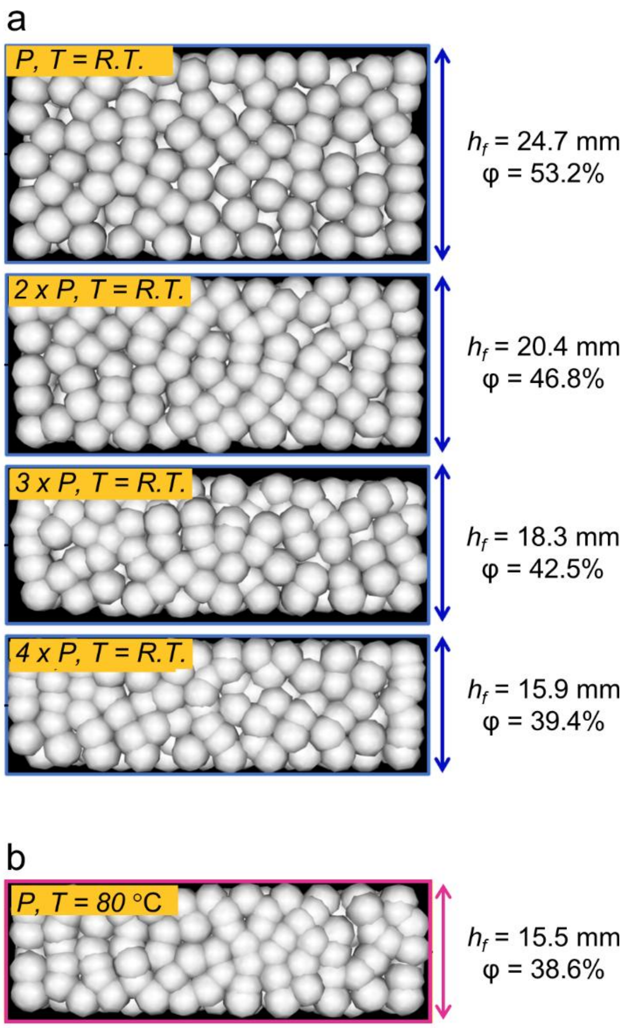

Figure S3. Effect of thermal expansion on computational compaction of sulfur powder beds. a) RT model and b) 80 °C model. (φ, porosity; h_i, initial powder bed height; h_f, final powder bed height; d_i, initial particle diameter; d_f, final particle diameter.)

Simulations employing various pressures between 50 MPa and 250 MPa reveal that a porosity of 39.3% can be obtained at 25 °C by applying a pressure of 200 MPa, which is four times the pressure required for the same porosity at 80 °C. These results highlight the importance of temperature in reducing the mechanical load required for densification and consolidation of powder bed in sulfur-based systems.

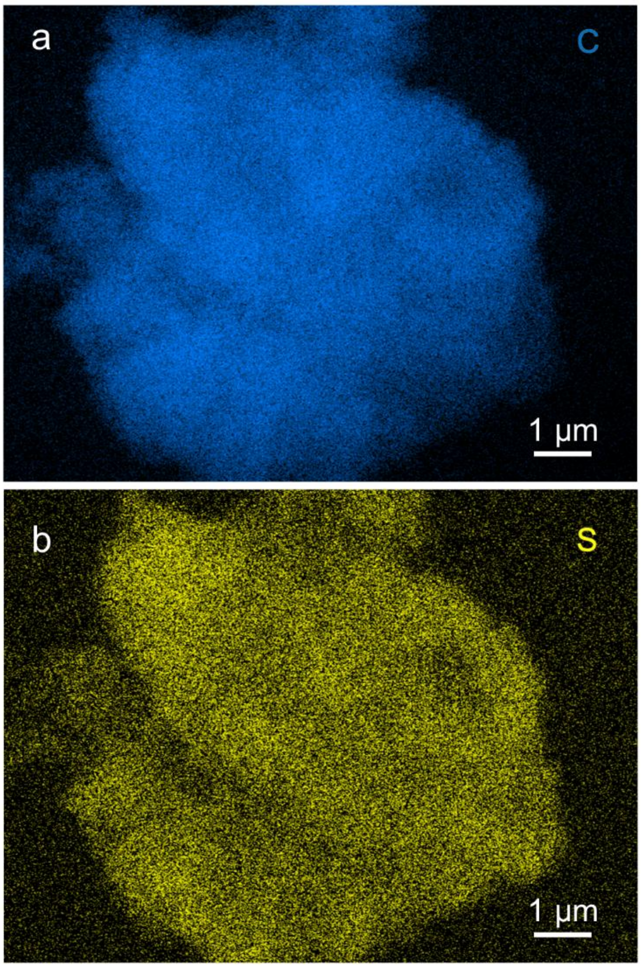

Figure S4. Scanning electron microscopy–energy dispersive X-ray spectroscopy elemental maps of the S-C composite particle shown in Fig. 2a. a) carbon and b) sulfur.

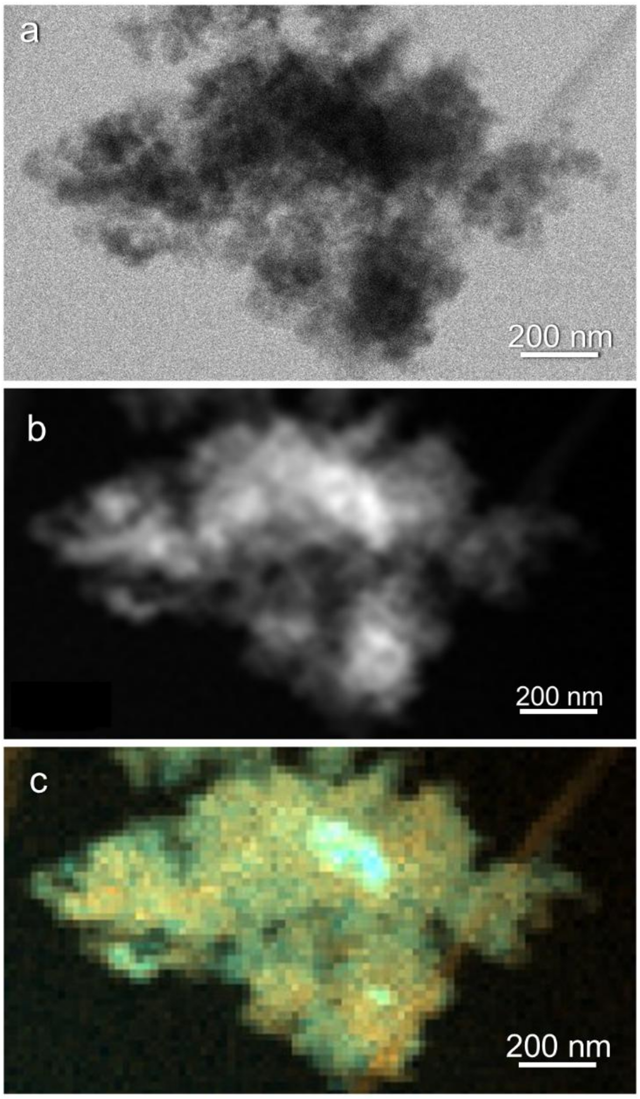

Figure S5. Transmission electron microscopy analysis results of an S-C composite. a) bright field, b) high-angle annular dark-field scanning transmission electron microscopy, and c) energy-dispersive X-ray spectroscopy elemental mapping (sulfur: yellow, carbon: blue) images of S-C composite particle, corresponding to Fig. 2b and 2c.

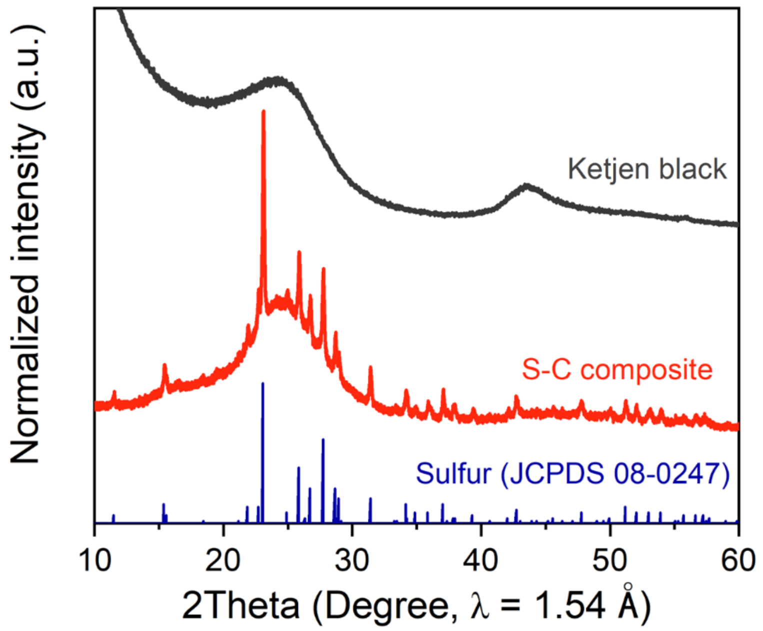

Figure S6. X-ray diffraction patterns of Ketjen black (black line), S-C composite powder (red line) and reference pattern of sulfur (blue line).

The pure Ketjen black shows broad peaks, indicating its amorphous nature. In contrast, the S-C composite exhibits prominent diffraction peaks corresponding to crystalline α-sulfur.

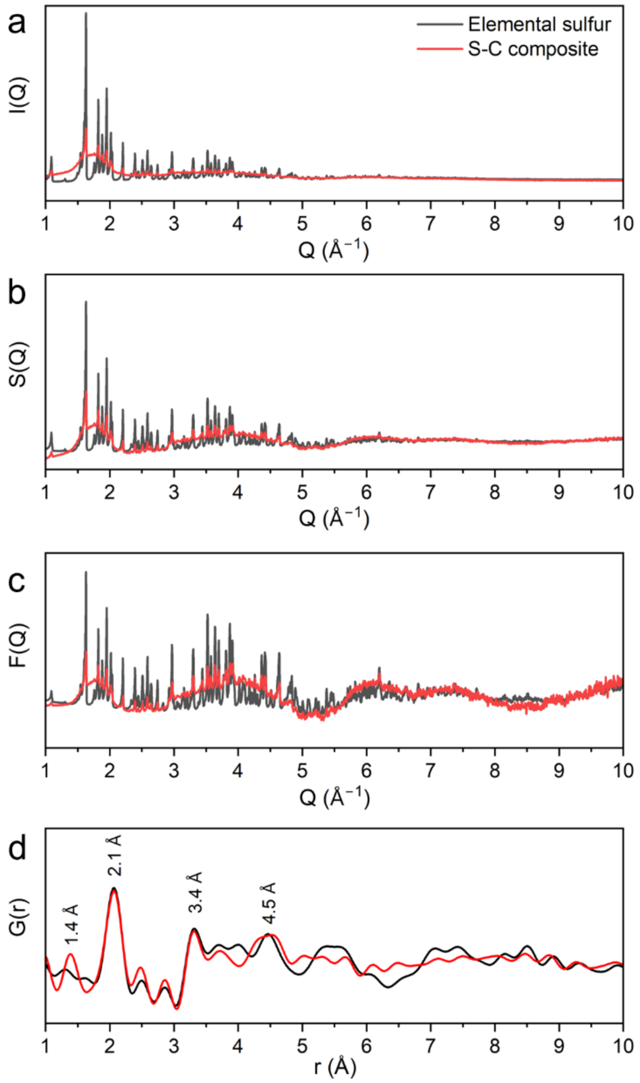

Figure S7 Pair distribution function (PDF) analysis comparing elemental sulfur (black) and the synthesized sulfur-carbon (S-C) composite (red): a) background-subtracted scattering intensity I(Q), b) total scattering structure function S(Q), c) reduced structure function F(Q), d) real-space PDF G(r).

The diffraction profiles of the melt-diffused S-C composite exhibit broadened features in I(Q) and S(Q) relative to the sharp Bragg peaks observed in elemental sulfur, particularly around q ≈ 1.7 Å^{-1}. This broadening signifies partial amorphization of sulfur as it diffuses into the porous carbon framework. Further evidence is provided by the real-space pair distribution function, G(r), where the melt-diffused sample displays an attenuation of long-range correlations beyond ~5 Å, in contrast to the extended order seen in elemental sulfur. Nevertheless, the characteristic nearest-neighbor S-S distances at 2.1 Å, 3.4 Å, and 4.5 Å are preserved, indicating the local S-S bonding is maintained. Additionally, a minor distance at 1.4 Å appears exclusively in the melt-diffused sample, which is attributed to the carbon framework in the S-C composite. These results collectively confirm the presence of amorphous sulfur domains formed through melt infiltration and confinement within the porous carbon matrix.

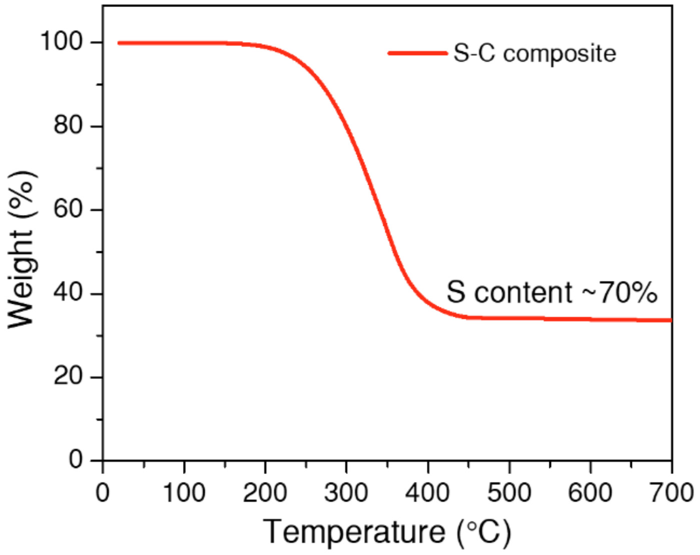

Figure S8. Thermogravimetric analysis result of sulfur-carbon (S-C) composite at the ramping rate of 5 °C min^{-1} under helium atmosphere.

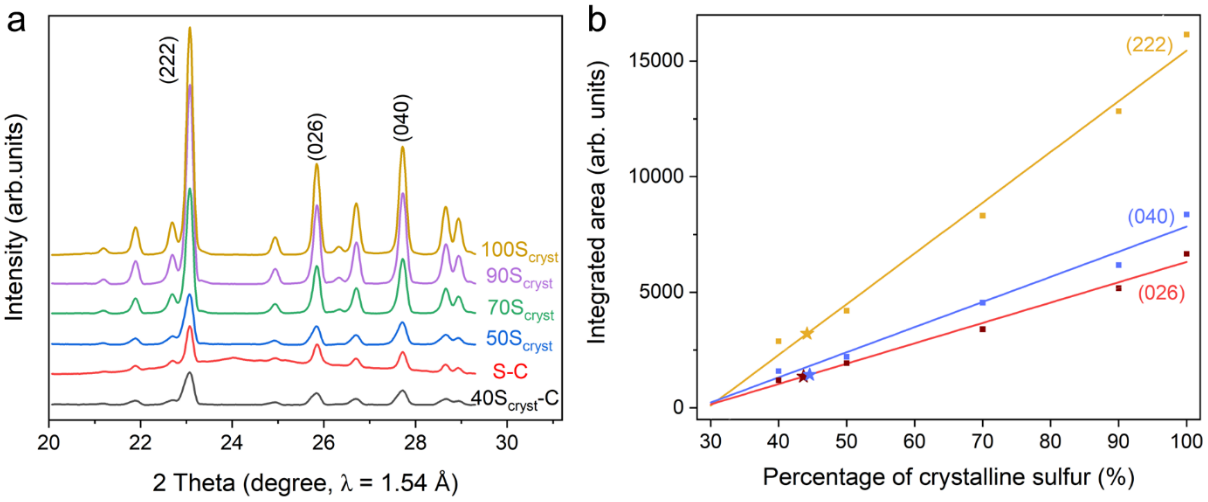

Figure S9. Quantification of crystalline sulfur fraction in the sulfur-carbon (S-C) composite X-ray diffraction (XRD) peak integration. (a) XRD patterns of reference crystalline sulfur, crystalline S-C mixtures (S_{cryst}-C) with varying sulfur ratios and the S-C composite. (b) Integrated peak areas of the (222), (026), and (040) reflections plotted against crystalline sulfur content. Symbols denote individual measurement (n = 1 per composition), solid lines represent linear regression fits (R^2 values of 0.97-0.99). Squares indicate S_{cryst}-C mixture and the stars denote the synthesized S-C composite.

The estimated crystalline sulfur fraction in the S-C composite is approximately 44 wt.%. XRD peak integration results are summarized in Table S5

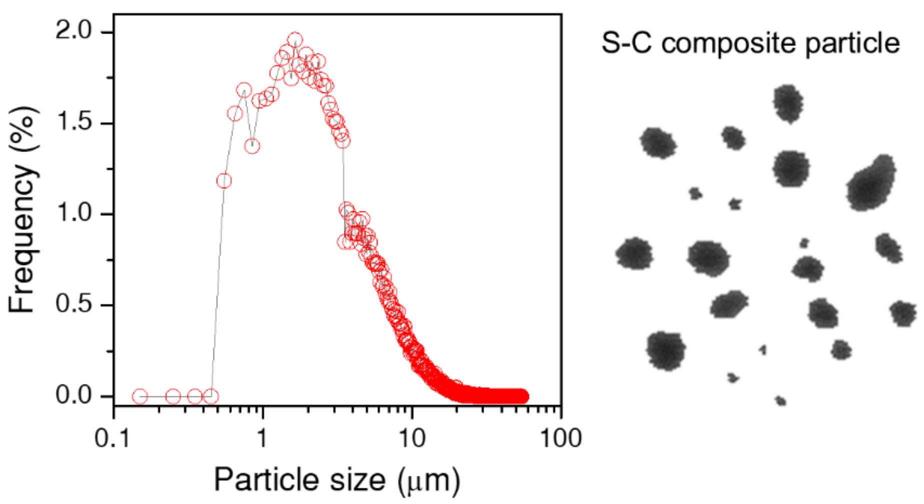

Figure S10. Particle size and morphology analysis of the sulfur-carbon composite.

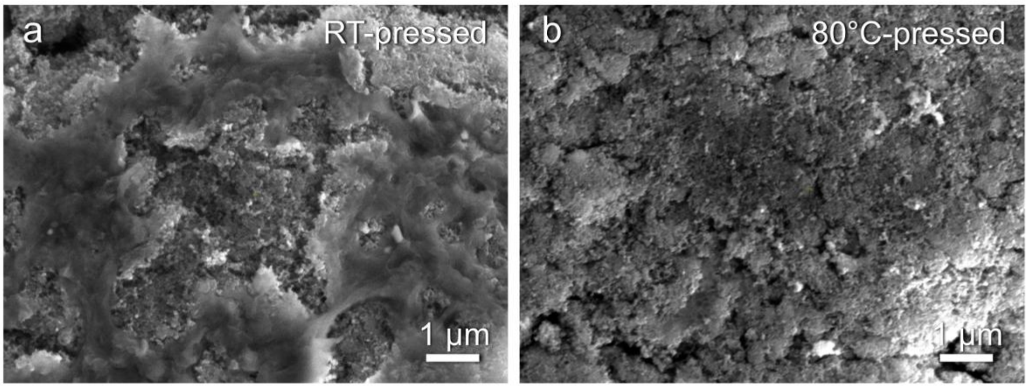

Figure S11. High magnification scanning electron microscope images of sulfur-carbon composite after the compression at a) room temperature (RT) and b) 80 °C.

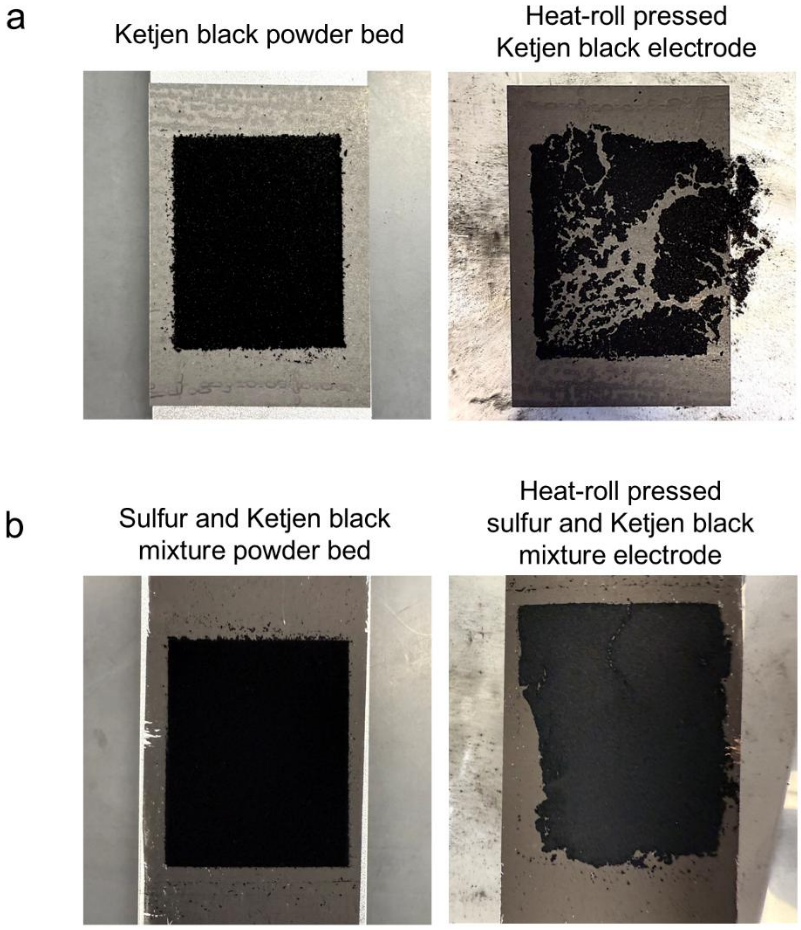

Figure S12. Photographs of the powder bed and the corresponding pressed electrode prepared at 80 °C. a) Ketjen black only and b) physical mixture of sulfur (70 wt.%) and Ketjen black (30 wt.%) powder bed.

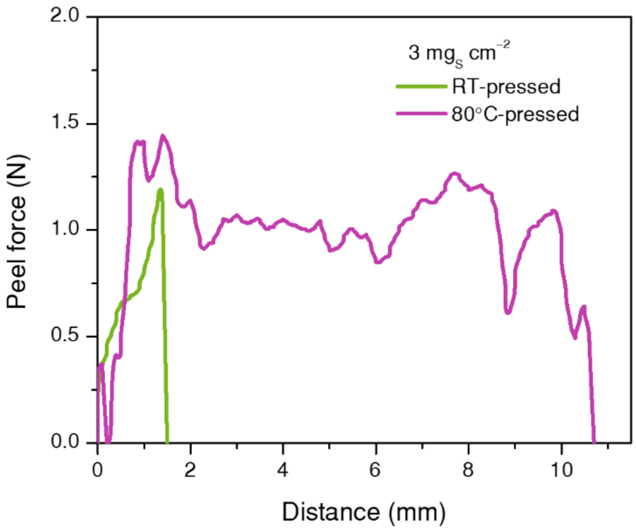

Figure S13. Peel force test results of the room temperature (RT)-pressed and 80 °C-pressed sulfur-carbon composite electrodes. The areal sulfur loading is 3 mg_S cm^{-2}.

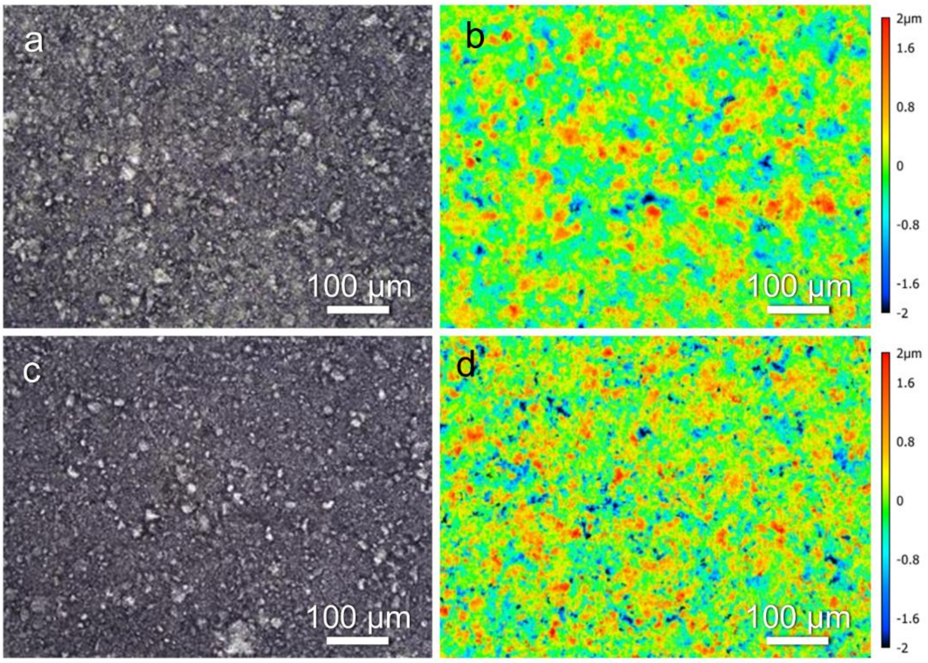

Figure S14. Surface morphology and topography of room temperature (RT)-pressed and 80 °C-pressed sulfur-carbon (S-C) composite electrodes. Top-view surface a) optical microscopy (OM) image and b) surface topology analysis of RT-pressed S-C composite electrodes, and c) OM image and d) surface profilometry analysis of 80 °C-pressed S-C composite electrodes. The areal sulfur loading is 3 mg_S cm^{-2}.

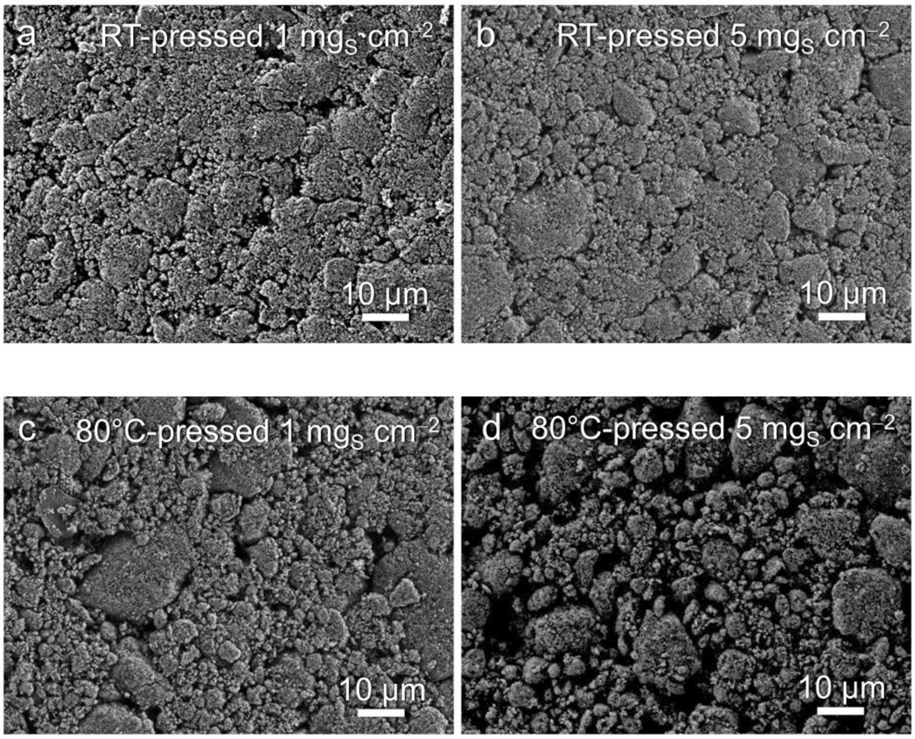

Figure S15. Surface morphology of binder-free sulfur-carbon composite electrodes with varying sulfur areal loadings pressed at room temperature (RT) and at 80 °C. Top-view scanning electron microscopy images of electrodes with areal sulfur loading of a) 1 mg_S cm^{-2} and b) 5 mg_S cm^{-2} pressed at RT, and c) 1 mg_S cm^{-2} and d) 5 mg_S cm^{-2} pressed at 80 °C.

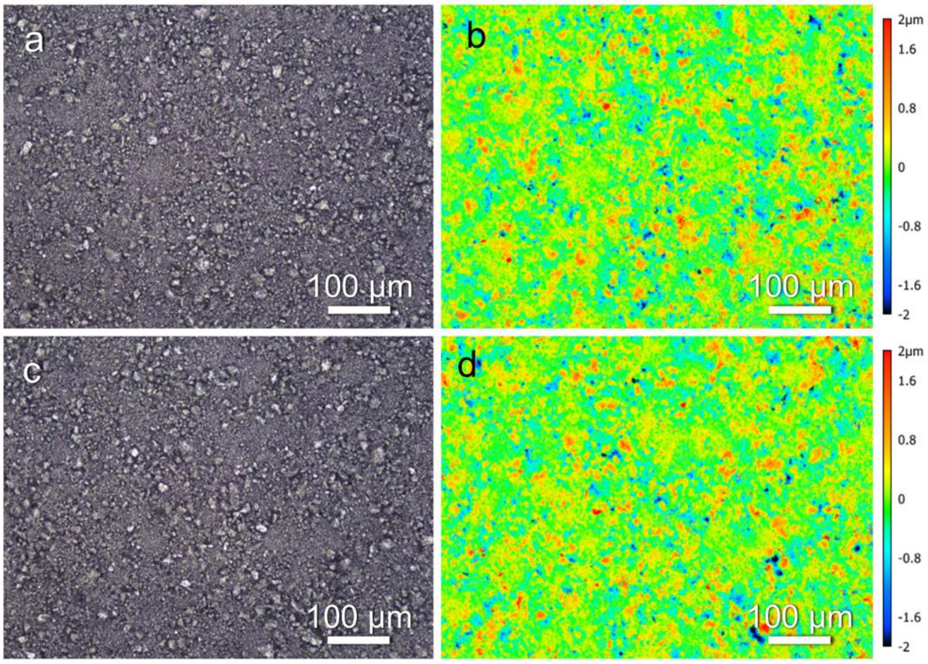

Figure S16. Surface morphology and topography of room temperature (RT)-pressed and 80 °C-pressed sulfur-carbon (S-C) composite electrodes. Top-view surface a) optical microscopy (OM) image and b) surface topology analysis of RT-pressed S-C composite electrodes, and c) OM image and d) surface profilometry analysis of 80 °C-pressed S-C composite electrodes. The areal sulfur loading is 1 mg_S cm^{-2}.

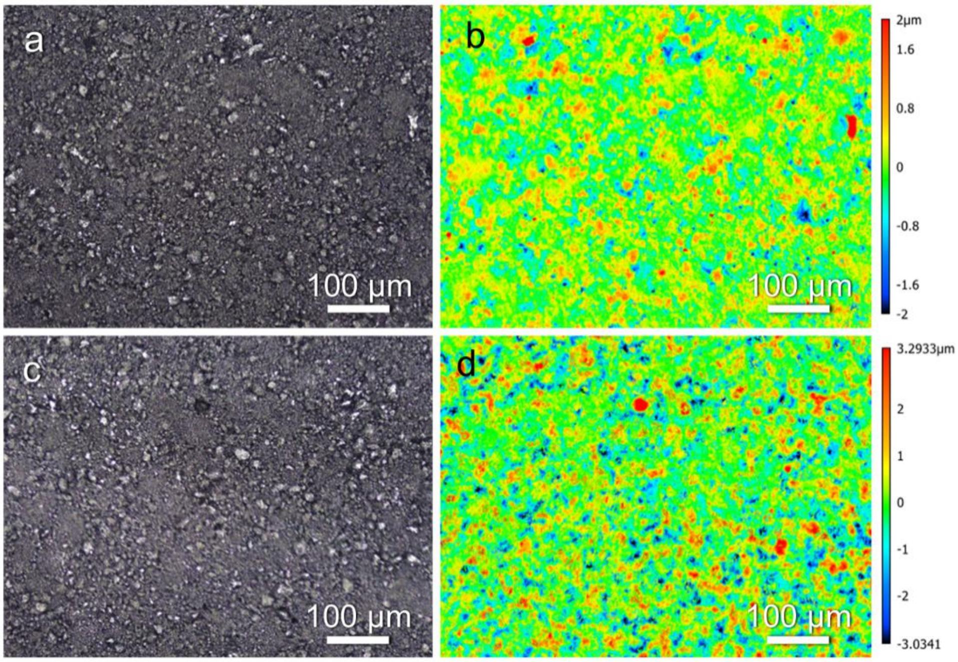

Figure S17. Surface morphology and topography of room temperature (RT)-pressed and 80 °C-pressed sulfur-carbon (S-C) composite electrodes. Top-view surface a) optical microscopy (OM) image and b) surface topology analysis of RT-pressed S-C composite electrodes, and c) OM image and d) surface profilometry analysis of 80 °C-pressed S-C composite electrodes. The areal sulfur loading is 5 mg_S cm^{-2}.

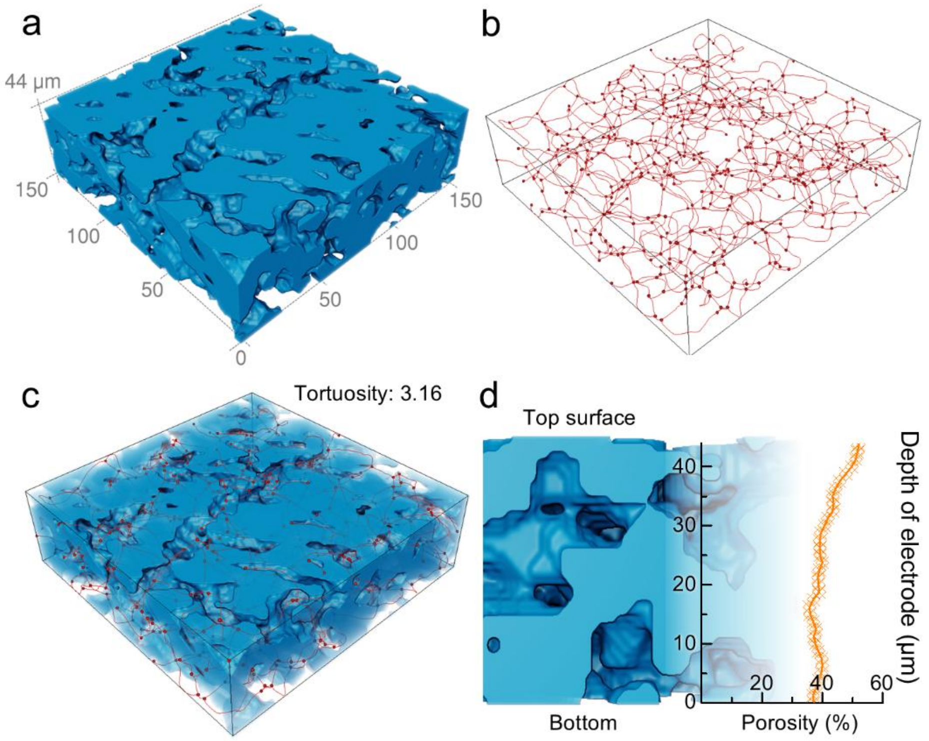

Figure S18. X-ray micro compute tomography (μ-CT) analysis results of the slurry-cast sulfur-carbon composite electrodes. a) X-ray μ-CT reconstructed three-dimensional (3D) image, b) skeleton nodes and intricate channels of tortuosity, c) skeleton nodes and channels on a 3D reconstruction overlaid image, and d) cross-sectional view of the reconstructed 3D image and its porosity map. The areal sulfur loading of the electrode is 3 $mg_S\ cm^{-2}$. The unit dimension is 150 μm × 150 μm × 44 μm.

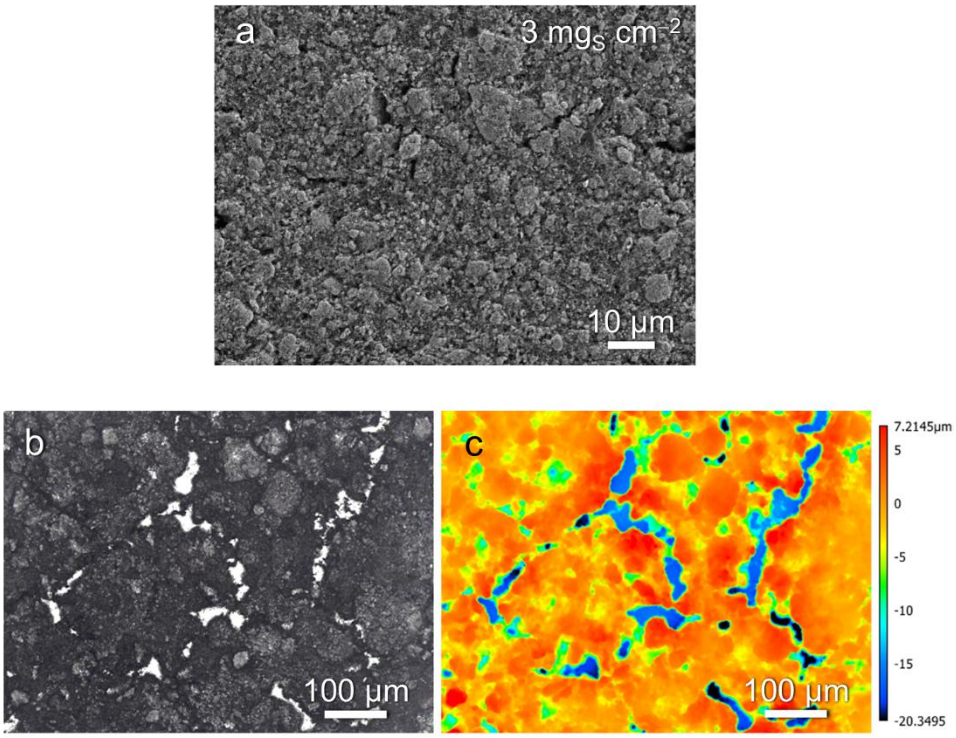

Figure S19. Surface morphology and topography of the slurry-cast sulfur-carbon (S-C) electrodes. Top view surface a) scanning electron microscopy and b) optical microscopy images with c) surface topology analysis of the slurry-cast S-C composite electrodes. Table S9 summarizes the physical properties of the slurry-cast S-C electrodes. The areal sulfur loading is 3 mg_S cm^{-2}.

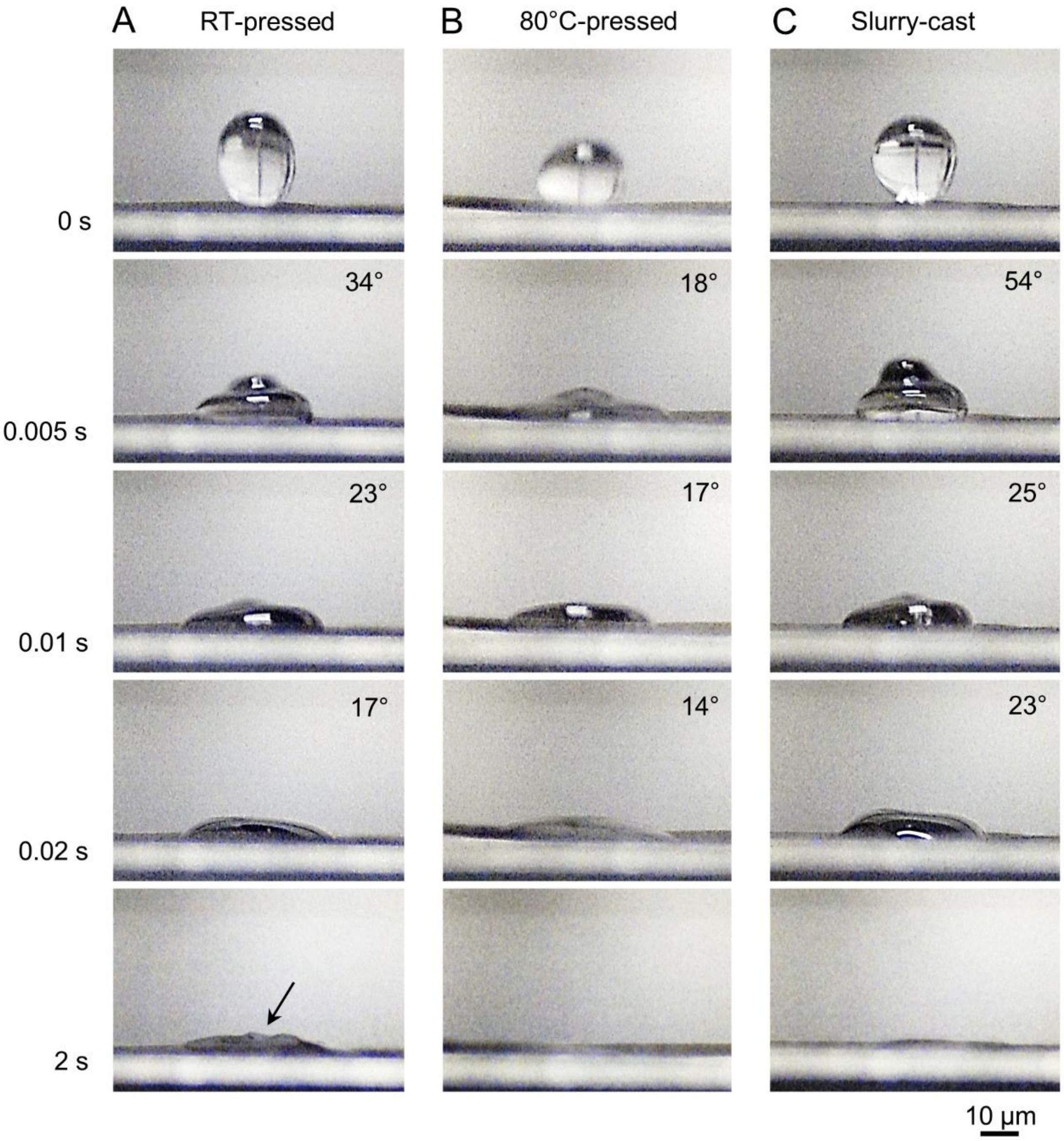

Figure S20. Electrolyte wetting test results on room temperature (RT)-pressed, 80 °C-pressed, and slurry-cast sulfur-carbon (S-C) composite electrodes. High-speed camera snapshots of electrolyte wetting on the a) RT-pressed, b) 80 °C-pressed, and c) slurry-cast S-C composite electrodes.

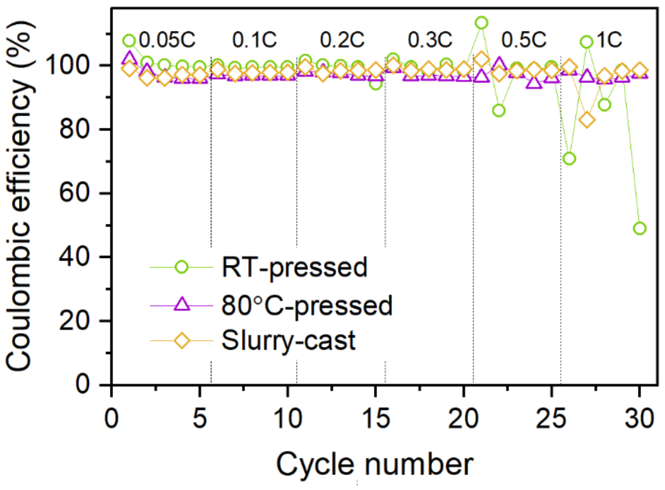

Figure S21. Coulombic efficiency during the rate-capability test at various C-rates (1C = 1675 mA g_S^{-1}, testing temperature: 25 °C), corresponding to the rate-performance data shown in Fig. 4a.

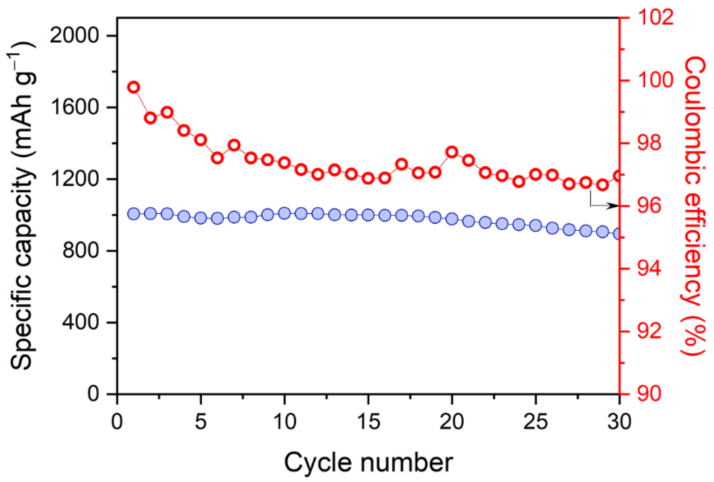

Figure S22. Charge-discharge cycling performance of a single-layer pouch cell using an 80 °C-pressed sulfur-carbon composite positive electrode. 1.5 × 1.5 cm^2 electrode with areal sulfur loading of 3 mg_S cm^{-2} was galvanostatically cycled at 0.3 C (1 C = 1675 mA gS^{-1}) within a cell voltage window of 1.7–2.7 V.

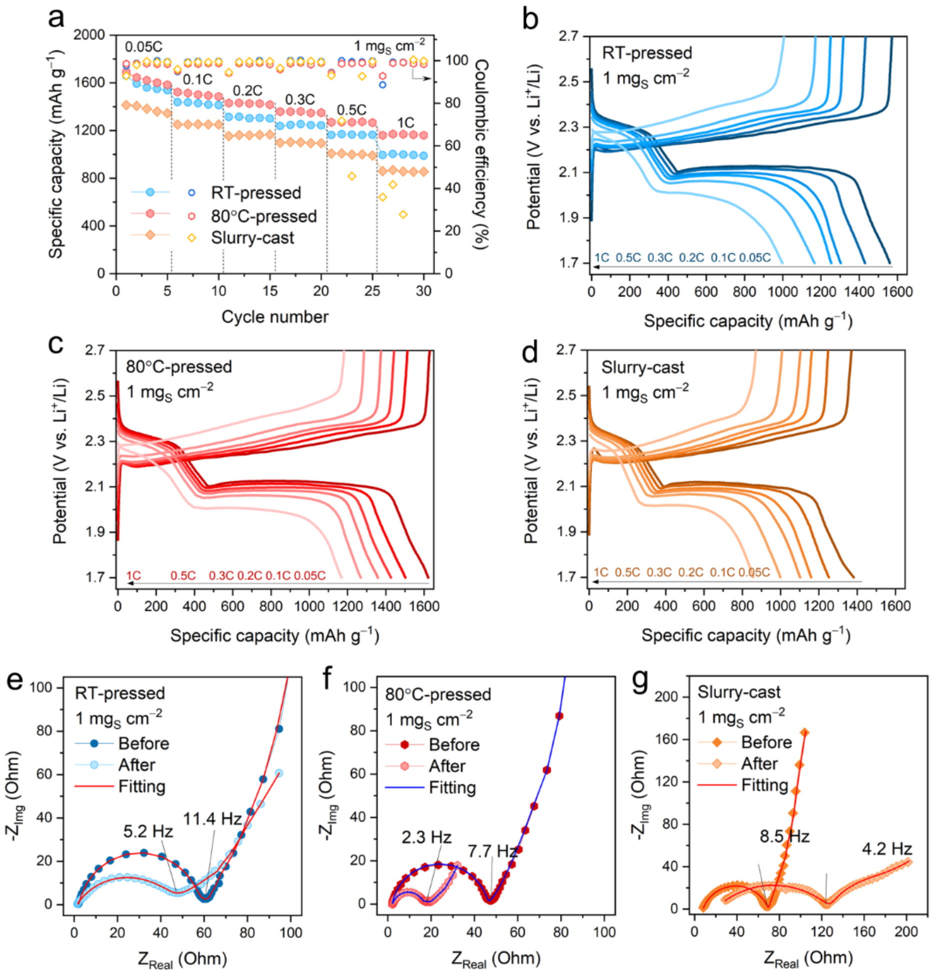

Figure S23. Electrochemical performance of sulfur-carbon (S-C) composite positive electrodes. a) Rate capability test results and voltage profiles of b) room temperature (RT)-pressed, c) 80 °C-pressed, and d) slurry-cast electrodes at various C-rate (1 C = 1675 mAh g_S^{-1}). Nyquist plot of e) RT-pressed, f) 80 °C-pressed, and g) slurry-cast electrodes, measured at the fully charged state before and after 30 cycles. Areal sulfur loading is 1 mg_S cm^{-2}.

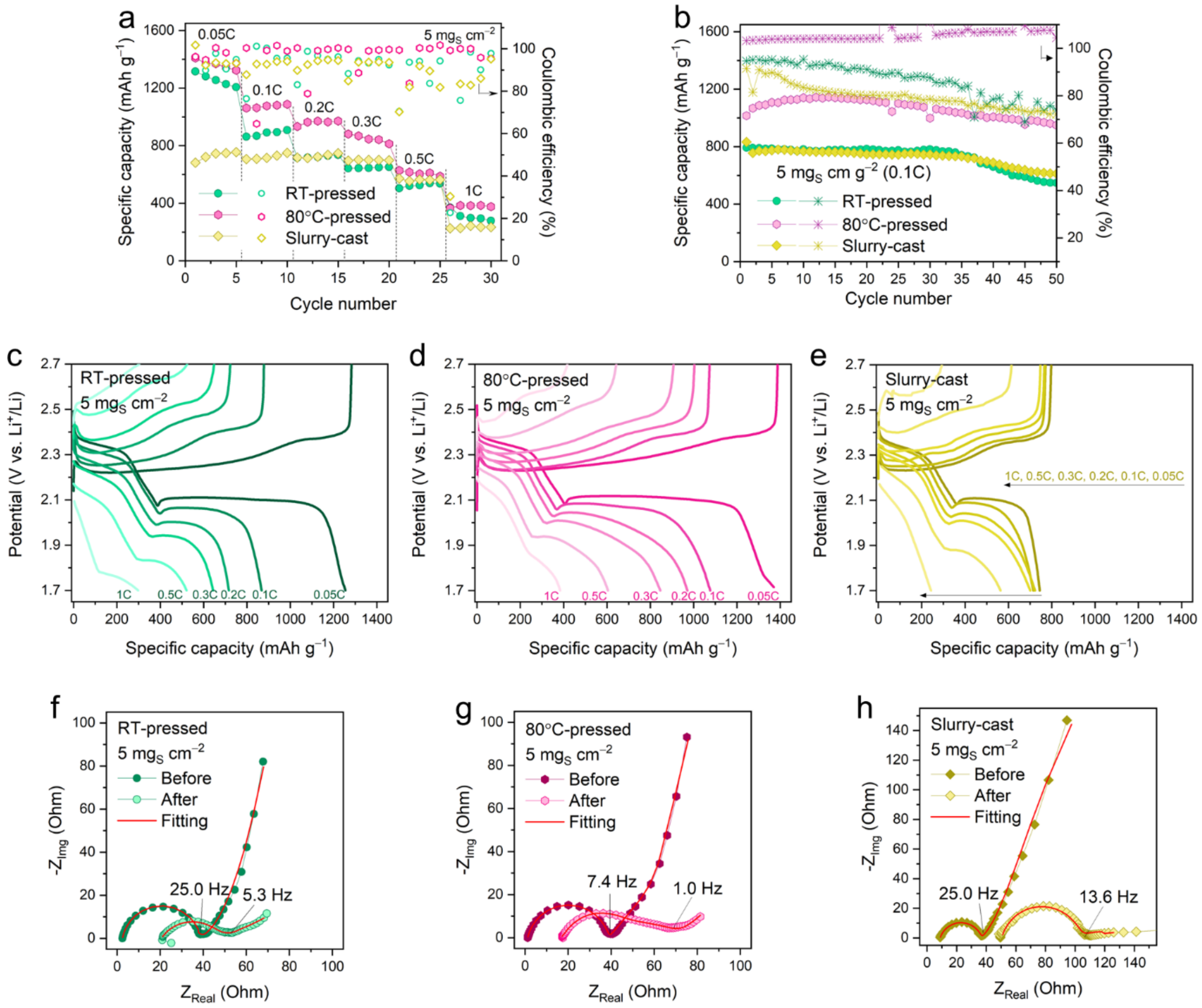

Figure S24. Electrochemical performance of sulfur-carbon (S-C) composite positive electrodes. a) Rate capability at various C-rates and b) cycling performance at 0.1 C (1 C = 1675 mAh g_S^{-1}). Voltage profiles of c) room temperature (RT)-pressed, d) 80 °C-pressed, and e) slurry-cast S-C electrodes during the rate capability test. Nyquist plot of f) RT-pressed, g) 80 °C-pressed, and h) slurry-cast S-C electrodes measured at the fully charged state before and after 50 cycles. Areal sulfur loading is 5 mg_S cm^{-2}.

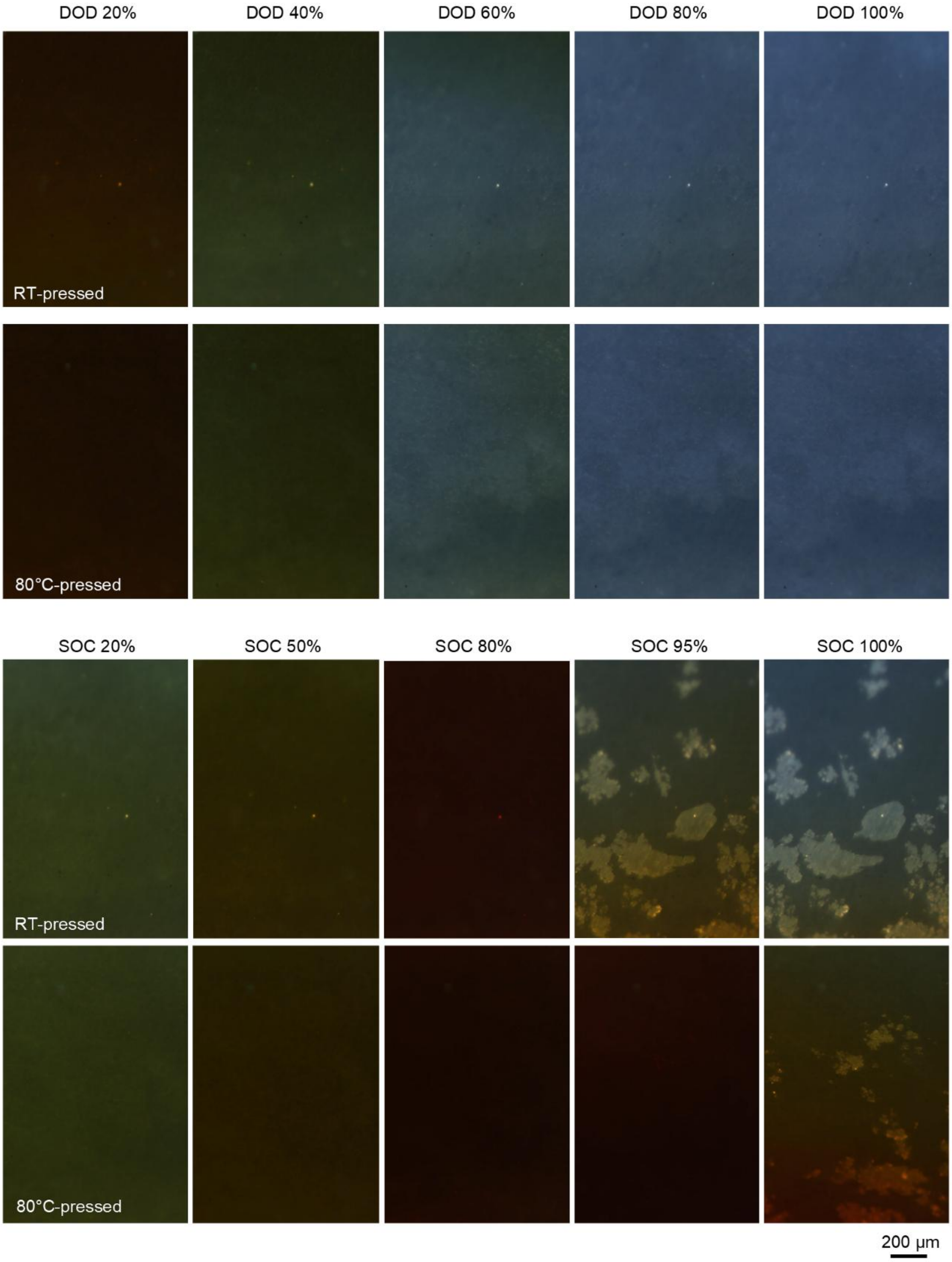

Figure S25. In situ optical microscopy (OM) images of room temperature (RT)-pressed and 80 °C-pressed electrodes at selected depths of discharge (DOD) and states of charge (SOC) during the first cycle. Electrochemical cells for in situ OM experiment was charged and discharged at 0.1 C within a cell voltage range of 1.8–2.7 V at 25 °C. OM images are acquired at various depths of discharge and states of charge during the first discharge and subsequent charge.

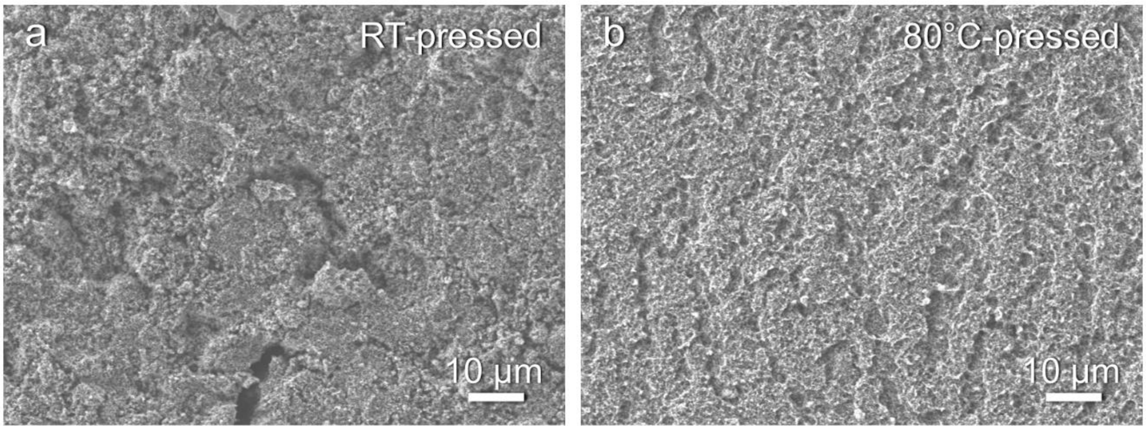

Figure S26. Structure change of room temperature (RT)-pressed and 80 °C-pressed sulfur-carbon positive electrodes during cycling. Ex situ scanning electron microscopy images collected at 40% depth of discharge during the initial discharge at C/20 at 25 °C (target capacity: 520 mAh g^{-1}, based on a discharge capacity of 1300 mAh g^{-1}). a) RT-pressed and b) 80 °C-pressed electrode.

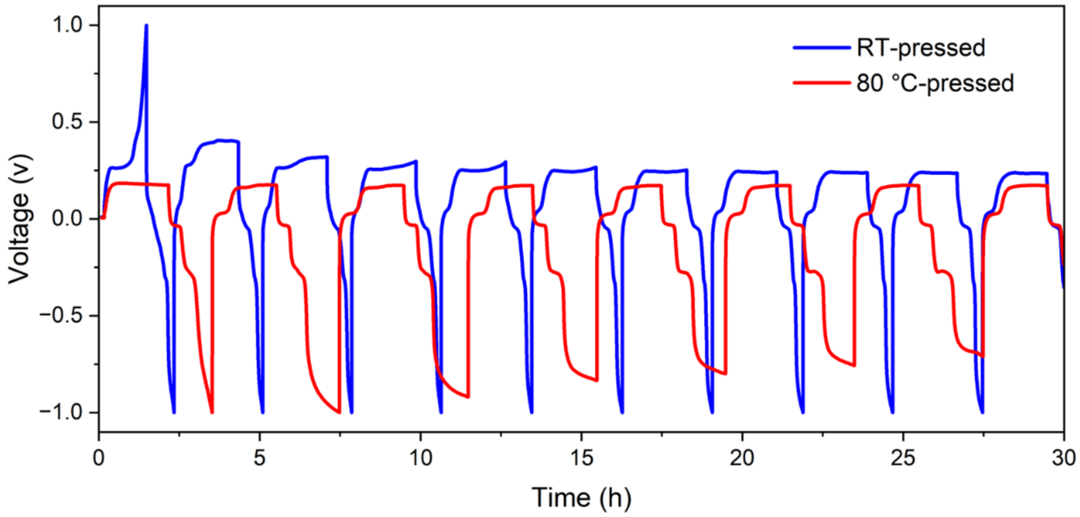

Figure S27. Galvanostatic cycling profiles of sulfur||lithiated sulfur (S||Li_2S) cells. An electrochemically lithiated sulfur electrode (Li_2S-containing) was paired with a fresh sulfur-carbon composite electrode. Cycling was conducted at 0.2 C (1 C = 1675 mAh g_S^{-1}) within −1 V to 1 V, with a 2 h cutoff per step at 25 °C.

Supplemental Tables

Table S1. Comparison of fabrication costs of sulfur-carbon (S-C) composite positive electrode for 1 kg of sulfur in the positive electrode.

	Cost ($/kg)	Our process		Slurry casting	
		Amount (kg)	Cost ($)	Amount (kg)	Cost ($)
A. Material Cost					
Elemental sulfur	0.4	1	0.4	1	0.4
Ketjen black	80	0.43	34.4	0.43	34.4
S-C composite (70 %S)	24.28	1.43	34.8	1.43	34.8
PVDF Binder	15	0	0	0.16	2.4
NMP	2.7	0	0	10.6	28.62
Aluminum foil	4	0.8	3.2	0.8	3.2
B. Process Cost					
Slurry mixing cost per kg of slurry	0.002	0	0	12.17	0.024
NMP drying & recovery cost per kg of NMP	1.12	0	0	10.6	11.87
Total Cost ($/kg of S)		38.40		80.91	

Material and energy costs except for the price of sulfur and ketjen black were estimated using the information in BatPac 5.0[S1] and a research paper.[S2] Following assumptions were made for estimate comparison. The following assumptions were made for estimating the cost.

1. The fabrication cost of the S-C composite is not considered for comparison as the same composite will be used for both processes.
2. Sulfur content in the S-C composite is 70 wt.%.
3. Binder content in slurry cast electrode is 10 wt.%.
4. Solid (S-C composite and poly(vinylidene fluoride (PVDF) binder) to NMP ratio is 0.15.
5. NMP drying & recovery cost per kg of NMP: $1.12
6. For slurry mixing, energy of 0.02 kWh/kg of slurry and $0.1/kWh of energy cost are considered
7. Energy cost for powder compaction of our process is not considered as slurry casting process also requires calendaring process utilizing the same principle.

Table S2. Summary of various sulfur host materials and electrochemical properties for Li||S batteries.

Active material for sulfur positive electrodes	Type of electrode processing	Binder	Current collector	Sulfur content of active material (%)	Sulfur loading (mg_S cm^{-2})	Capacity at initial / 100th cycle / Subsequent cycles (mAh g^{-1})	Ref.
**S-C composite**	**Solvent-free direct active casting**	**No binder**	**Al foil**	**70**	**1.0**<br>**3.0**	**1180 / 1127 / 932 after 500 cycles 1 C**<br>**1280 / 1035 / 780 after 200 cycles at 0.2 C**	**This study**
S/CNT composite with single-atom nickel-CNTs/PP separator	Slurry-cast	10 wt.% LA133	Al foil	80	1.5<br>4.0	1008 / 900 / 726 after 500 cycles at 1 C<br>1000 / 800 / 750 after 200 cycles at 0.2 C	S3
S/C composite with single-atom cobalt carbon interface	Slurry-cast	10 wt.% LA133	Al foil	70.2	1.2<br>3.0	1014 / 1000 / 781 after 500 cycles at 1 C<br>1004 / 880 / 870 after 100 cycles at 0.1 C	S4
S/C composite with Co coated separator interlayer	Slurry-cast	10 wt.% LA133	Al foil	65	1.5<br>5.2	1100 / 1000 / 910 after 100 cycles at 0.5 C<br>1050 / 750 / 710 after 100 cycles at 0.2 C	S5
S@Pt composite with carbon nanofiber layer	Slurry-cast	10 wt.% Na alginate binder	Al foil	85	1.2<br>2.7	1100 / 850 / 737 after 350 cycles at 1 C<br>1227 / 981 after 65 cycles at 0.3 C / -	S6
S/C composite	Slurry-cast	10 wt.% GN-BA	Al foil	49.9	2.1<br>5.0	1140 / 752 after 100 cycles at 0.2 C / -<br>1140 / 627 after 100 cycles at 0.2 C / -	S7
SeS_2 on AvCarb P50 carbon paper	Slurry-cast	10 wt.% PVDF	Carbon paper	56.1[a]	2.0	920 / 700 after 100 cycles at 0.5 C / -	S8
S/CNT with Ti-Metal organic frameworks-Graphene	Slurry-cast	10 wt.% PVDF	Al foil	70.3	2.1<br>4.5	1095 / 1000 / 898 after 500 cycles at 1 C<br>1533 / 1482 after 100 cycles at 0.2 C / -	S9
Sublimed S with catalytic layer of V_2O_5/CMF	Slurry-cast	10 wt.% PVDF	Al foil	80	2.5	1000 / 876 after 100 cycles at 1 C / -	S10
S/NiCo nano composite	Slurry-cast	10 wt.% PVDF	Al foil	70	3.0<br>4.5	1050 / 750 / 620 after 200 cycles at 0.2 C<br>900 / 650 / 585 after 200 cycles at 0.2 C	S11
S/C composite	Slurry-cast	5 wt.% Tragacanth	Al foil	61.7	1.1–1.4	1188 / 813 / 820 after 300 cycles at 1 C	S12
S/C composite	3D-print using S/C slurry ink	2 wt% PVDF-HFP	No CC	42[a]	3.0<br>5.5	1184 / 820 / 752 after 200 cycles at 0.2C<br>1188 / 750 / 606 after 200 cycles at 1 C	S13

Active material for sulfur positive electrodes	Type of electrode processing	Binder	Current collector	Sulfur content of active material (%)	Sulfur loading (mg_S cm^{-2})	Capacity at initial / 100th cycle / Subsequent cycles (mAh/g)	Ref.
Mo_2C-coated CNT/CF film	Chemical deposition	No binder	CNT/CF film	62.5	1.0	1100 / 990 / 800 after 500 cycles at 1C	S14
S/Carbon composite	Dry-cast	No binder	Expanded metal	66.7[a]	4.5	850 / 800 after 30 cycles at 0.1C	S15
S/carbon composite	Dry-cast	1 wt.% PTFE	Al foil	64	8.0	651 / 513 after 80 cycles at 0.05C / -	S16
Se-doped sulfurized polyacrylonitrile (Se-SPAN)	Dry-cast	10 wt.% PTFE	Carbon coated Al foil	52.2[a]	3.26[a]	615 / 580 after 100 cycles at 0.2 A $g_{Se\text{-}SPAN}^{-1}$	S17

[a]This value is calculated by the authors.

Table S3. XRD peak shift values derived from the operando XRD data presented in Fig. 1a and S1.

Roll pressed temperature (°C)	Peak shifting, 2Theta (Degree, λ = 0.56 Å)						
	(222)	(133)	(026)	(311)	(040)	(313)	(135)
Room temperature	8.325	8.985	9.315	9.615	9.975	10.320	10.420
40	8.325	8.985	9.315	9.600	9.975	10.300	10.410
60	8.310	8.985	9.300	9.600	9.960	10.300	10.390
80	8.300	8.970	9.300	9.585	9.960	10.290	10.390

Table S4. Rheological properties of the elemental sulfur measured at RT and 80 °C, estimated from the results shown in Fig. S3.

Elemental sulfur		
Test temperature (°C)	Room temperature	80
Particle diameter (μm)	Few	Few
Cohesion at 15.6 kPa	0.85	1.03
Flow function	3.94	3.01

The estimated numerical properties of elemental sulfur at RT and 80 °C, corresponding to the Mohr's circle shown in Fig. 1b are summarized in Table S4. Cohesion measures the internal bonding strength between particles in a material. In powder rheology, it represents how well the particles stick together. Higher cohesion values indicate stronger interparticle forces, which can result from surface interactions or bonding. The flow function is a ratio used to describe a material's flowability. A higher flow function indicates easier flowability, meaning the material can flow more readily under applied stresses. Conversely, a lower flow function suggests that the material is more prone to sticking or clumping, which makes the flow more difficult. Mohr's circles shown in Fig. 1b illustrate the relationship between applied normal stress and shear stress for elemental sulfur at RT and 80 °C. Each circle represents the stress state of sulfur under different conditions of applied normal stress during the powder rheology test. The smaller circles

correspond to lower normal stress, while the larger circles represent higher normal stress applied. The increased size of the blue circles (RT) compared to the red circles (80 °C) at higher stresses indicates that sulfur exhibits greater resistance to shear stress and less softening at RT. In contrast, at 80 °C, sulfur undergoes more pronounced softening, as shown by the smaller red circle at higher normal stress. The higher cohesion (y-axis intercept) and reduced shear resistance at 80 °C suggest stronger interparticle bonding but lower overall strength, likely due to thermal softening or partial sintering. This softening at elevated temperatures causes sulfur particles to deform more easily, contributing to lower flowability and increased particle bonding.

Table S5. XRD Peak integration results of S_{cryst}-C and the S-C composites

	**(222)**		**(026)**		**(040)**	
Sample	FWHM (°)	Integrated area (a.u.)	FWHM (°)	Integrated area (a.u.)	FWHM (°)	Integrated area (a.u.)
100S_{cryst}	0.16	16148	0.16	8367	0.16	6662
90S_{cryst}-C	0.16	12835	0.16	6178	0.16	5174
70S_{cryst}-C	0.16	8312	0.16	4554	0.16	3402
50S_{cryst}-C	0.16	4203	0.16	2210	0.16	1935
40S_{cryst}-C	0.16	2882	0.16	1193	0.16	1590
S-C	0.17	3210	0.17	1433	0.17	1354

Table S6. Summary of particle size and morphology analysis results for the S-C composite**.**

S-C composite				
D10	D50	D90	Aspect ratio	Circularity
7.07	14.9	30.4	0.974	0.896

D10, D50, and D90 represent particle size distribution percentiles. DX means X% of the particles are smaller than the measured value. The aspect ratio is the ratio of the length of the particle to its width. A value closer to 1 indicates the particle is more equiaxed (or spherical), while values far from 1 suggest elongated or flattened shapes. Circularity is a measure of how close the shape of a particle is to a perfect circle. A value of 1 indicates a perfect circle, while values less than 1 indicate more irregular shapes.

Table S7. Rheological properties of the S-C composite measured at RT and 80 °C, estimated from the results shown in Fig. 2f.

S-C composite		
Test temperature (°C)	Room temperature	80
Particle diameter (μm)	30	30
Cohesion at 15.6 kPa	0.8	0.88
Flow function	5.36	5.25

Table S8. Physical properties of the prepared S-C composite positive electrodes at RT and 80 °C. Bulk porosity was calculated based on the measured areal mass loading and the known densities of sulfur and carbon. Surface void percentage was estimated using SEM images shown in Fig. 3c and 3f.

Type of S-C composite electrode	Sulfur mass (mg_S cm^{-2})	Surface void (%)	Bulk porosity (%)	Surface roughness S_{pc} (mm^{-1})
RT-pressed	1	39	52.2	1451.9
	3	40	54.1	1192.0
	5	44	51.9	992.25
80 °C-pressed	1	42	53.5	1420.3
	3	49	56.2	1612.7
	5	65	56.5	2286.7

Table S9. Physical properties of the slurry-cast S-C composite positive electrodes with an areal mass loading 3 mg_S cm^{-2}.

	Surface void (%)	Bulk porosity (%)	Surface roughness S_{pc} (mm^{-1})
Slurry-cast	23	53.3	1197.5

Table S10. Fitted parameters extracted from the equivalent circuit model shown in Fig. 4l, corresponding to the Nyquist plots in Fig. 4i–k, Fig. S22e–g, and Fig. S23f–h.

Electrode	Areal mass (mg_S cm^{-2}) & Cell state	R_s (Ω)	CPE_f (μF)	R_f (Ω)	CPE_{ct} (F)	R_{ct} (Ω)	W_o (Ω $s^{-1/2}$)	$\chi^2/\lvert Z\rvert$
RT-pressed	1.0, before	1.99	2.04	59.32	4.22×10^{-2}	3.10	70.2	0.113
	1.0, after	1.25	9.17	46.3	1.17×10^{-2}	12.1	17.5	0.082
	3.0, before	4.16	2.11	33.0	1.64×10^{-1}	12.1	35.4	0.082
	3.0, after	42.1	1.73	24.7	7.16×10^{-4}	21.5	3.61	0.016
	5.0, before	2.47	2.28	37.3	3.45×10^{-2}	3.68	11.0	0.085
	5.0, after	19.5	3.14	22.4	3.60×10^{-1}	44.1	10.9	0.031
80 °C-pressed	1.0, before	2.08	2.08	44.7	9.53×10^{-3}	0.16	35.7	0.064
	1.0, after	2.26	2.31	14.5	3.99×10^{-4}	1.97	3.70	0.089
	3.0, before	2.25	3.82	30.6	1.48×10^{-2}	1.08	21.48	0.059
	3.0, after	5.24	3.35	8.40	3.32×10^{-6}	8.47	2.23	0.024
	5.0, before	1.24	2.51	36.6	7.56×10^{-3}	7.13	21.7	0.011
	5.0, after	16.5	2.78	20.9	1.75×10^{-4}	35.26	2.53	0.019
Slurry-cast	1.0, before	7.90	1.99	60.1	3.88×10^{-2}	3.64	8.48	0.027
	1.0, after	19.4	1.95	110.2	3.00×10^{-2}	64.2	9.31	0.013
	3.0, before	5.76	3.37	34.5	1.20×10^{-4}	7.13	1.70	0.030
	3.0, after	45.41	2.37	35.1	9.20×10^{-4}	2.64	7.86	0.029
	5.0, before	8.83	2.92	28.6	3.66×10^{-2}	8.98	10.1	0.031
	5.0, after	49.6	2.81	48.5	6.14×10^{-2}	36.41	3.11	0.019

Supporting References

[S1] Knehr, K., Kubal, J., Nelson, P. & Ahmed, S. Battery performance and cost modeling for electric-drive vehicles: BatPaC v5.0 (US Department of Energy, 2022).

[S2] Ahmed, S., Nelson, P. A., Gallagher, K. G. & Dees, D. W. Energy impact of cathode drying and solvent recovery during lithium-ion battery manufacturing. J. Power Sources 322, 169–178 (2016).

[S3] Wu, Z. *et al.* Unveiling the autocatalytic growth of Li2S crystals at the solid-liquid interface in lithium-sulfur batteries. *Nat. Commun.* 15, 9535 (2024).

[S4] Li, Y. *et al.* Modulating the coordination environment of Co single-atom catalysts: Impact on lithium-sulfur battery performance. *Adv. Funct. Mater.* 35, 2412279 (2025).

[S5] Song, *Y. et al.* Seeding Co atoms on size effect-enabled V2C MXene for kinetically bosted lithium-sulfur batteries. *Adv. Funct. Mater.* 34, 2409748 (2024).

[S6] Gao, M. *et al.* Outstanding long-cycling lithium-sulfur batteries by core-shell structure of S@Pt composite with ultrahigh sulfur content. *Adv. Powder Mater.* 1, 100006 (2022).

[S7] Sun, R. *et al.* Water-soluble cross-linking functional binder for low-cost and high-performance lithium-sulfur batteries. *Adv. Funct. Mater.* 31, 2104858 (2021).

[S8] Kim, J. H. *et al.* Understanding the electrochemical processes of SeS_2 positive electrodes for delveloping high-performance non-aqueous lithium sulfur batteries. *Nat. Commun.* 15, 7669 (2024).

[S9] Guo, S. *et al.* Catalytic multivariable metal-organic frameworks for lithium-sulfur batteries. *Mater. Today* 65, 37−46 (2023).

[S10] Yang, B. Z. *et al.* Binder-free ω-$Li_3V_2O_5$ catalytic network with multi-polarization centers assists lithium-sulfur batteries for enhanced kinetics behavior. *Adv. Funct. Mater.* 32, 2110665 (2022).

[S11] Zhao, C. *et al.* Development of synergistically efficient Ni-Co pair catalytic sites for enhanced polysulfide conversion in lithium-sulfur batteries. *Adv. Funct. Mater.* 34, 2402175 (2024).

[S12] Senthil, C., Kim, S.-S. & Jung, H. Y. Flame retardant high-power Li-S flexible batteries enabled by bio-macromolecular binder integrating conformal fractions. *Nat. Commun.* 13, 145 (2022).

[S13] Gao, X. *et al.* Toward a remarkable Li-S battery via 3D printing. *Nano Energy* 56, 595−603 (2019).

[S14] Um, K. *et al.* Janus architecture host electrode for mitigating lithium-ion polarization in high-energy-density Li-S full cells. *Energy Environ. Sci.* 17, 9112–9121 (2024).

[S15] Horst, M. *et al.* A binder-free dry coating process for high sulfur loading cathodes of Li-S batteries: A proof-of-concept. *J. Power Sources* 587, 233675 (2023).

[S16] Sul, H., Lee, D. & Manthiram, A. High-loading lithium-sulfur batteries with solvent-free dry-electrode processing. *Small* 20, 2400728 (2024).

[S17] Kim, D.J. *et al.* Solvent-free dry-process enabling high-areal loading selenium-doped SPAN cathodes toward practical lithium-sulfur batteries. *Small* 21, 2503037 (2025).
