# Supplementary material for: Binding properties of sulfur to enable solvent-free fabrication of high-performance polymer-free sulfur-carbon positive electrodes
Source: Nat Commun. 2026 Feb 4;17:2360. doi: 10.1038/s41467-026-69097-6 (PMC12979846; doi:10.1038/s41467-026-69097-6)
Supplement: Supplementary file 2 — Description of Additional Supplementary Files [file 41467_2026_69097_MOESM2_ESM.pdf]

Title: Supplementary Movie 1.

Description: In situ optical microscopy (OM) of RT-pressed S-C cathode. Upper panel: galvanostatic voltage profile at 0.2 C (1 C rate corresponds to 1675 mA g_S^{-1}) at 25 °C plotted against specific capacity (mAh g_S^{-1}). The red marker on the curve indicates the instantaneous time point corresponding to the OM frame shown below. Lower panel: in situ OM image sequence of the cathode surface during cycling Scale bar: 200 μm.

Title: Supplementary Movie 2.

Description: In situ optical microscopy (OM) of 80 °C-pressed S-C cathode. Upper panel: galvanostatic voltage profile at 0.2 C (1 C rate corresponds to 1675 mA g_S^{-1}) at 25 °C plotted against specific capacity (mAh g_S^{-1}). The red marker on the curve indicates the instantaneous time point corresponding to the OM frame shown below. Lower panel: in situ OM image sequence of the cathode surface during cycling Scale bar: 200 μm.
